# Reflections on my experiences as a non-autistic autism researcher

**DOI:** 10.1177/13623613221121432

**Published:** 2022-09-02

**Authors:** Sandy Thompson-Hodgetts

**Affiliations:** University of Alberta, Canada

**Keywords:** collaboration, participatory research, positionally, reflexivity

I have been a clinician for over two decades and an autism researcher for almost as long. I also teach graduate-level courses related to autism. Early in my career, I did not think about being non-autistic. I never thought about whether my students, colleagues, conference attendees, or the people reading my manuscripts were autistic. I did not consider my identity as a non-autistic autism researcher.

Over the past decade or so, I have matured into my role as an autism researcher and educator. I have met more and more autistic colleagues and friends, read more and more work written by autistic people, and learned about novel (to me) participatory research methods. I have also worked to become an authentic autistic ally, actively challenging autistic oppression, through my research, teaching, and service. This was a shift from my early career.

A couple of years ago, we engaged a team of autistic adults as co-researchers on a nationally funded research project. We meet regularly about the project and receive feedback and input on all aspects of the study. There were many times when I thought that something was crystal clear, only to receive different feedback from this team. There was even a time when carefully chosen wording was interpreted so differently from my intention that some team members thought it could be deemed offensive. This was an important realization that the work I produced might not resonate with some of the autistic community in the way I intended.

I do not know if these differences in interpretation were something to do with my non-autistic brain or our different lived experiences, but research has shown that the lived experience of being autistic often offers unique insights and perspectives into things that a non-autistic person might not perceive (Finch et al., 2022; Gillespie-Lynch et al., 2017). Many autistic people, including my colleagues, students and friends, report experiences of conforming to others expectations and camouflaging what are perceived as autistic characteristics in an attempt to avoid marginalization and discrimination, which can challenge one’s self-perception and well-being (Finch et al., 2022; Hull et al., 2017). While I certainly acknowledge some discrimination in my life because I identify as a woman, I have not otherwise felt marginalized or stigmatized. I have almost always felt that I had a respected voice at the table. Autistic people have also long been faulted for social deficits, yet research has shown that communication between autistic people is as effective as communication between non-autistic people (Crompton et al., 2020). This knowledge is humbling as someone who long subscribed to the *DSM* diagnostic criteria that has centered autism around social deficits. Social situations are mutually and dynamically constructed, and I now realize that some of my research and teaching has reinforced neurotypical social norms as optimal.

Some autistic scholars discuss potential risks of non-autistic “experts” who teach about autism (e.g. Walker, 2021, p. 100). I am regarded as an autism “expert” by many students, colleagues, and others, but on what grounds? For example, can I adequately interpret qualitative data gathered from autistic research participants? I think there is value in, and space for, autistic and non-autistic autism researchers, but the collaborative experiences that I have had with my autistic colleagues, students, and friends have challenged my professional knowledge and expertise and made me reflect on what else I could do as an authentic autistic ally. I think that there are necessary responsibilities for me, and other, non-autistic people engaged in autism research.

The first step that I believe every non-autistic autism researcher should take is to become aware of one’s own positionality as an autism researcher and be critically reflexive about this, potentially new-found, knowledge. This is hard work and requires vulnerability. This is also a process that takes time and will be ongoing. Of course, this process also goes beyond one’s identity as non-autistic but that needs to be part of the process. As one, of many, examples, I was trained as a health-care professional and worked clinically with autistic children and their families for years, both primarily situated within a medical model. Acknowledging the biases associated with these roles and how they aligned with my beliefs about diversity and disability were critical to evolving my research program to where it is today, including the research methods I engage, which is vastly different from where it started.

Second, I recommend engaging autistic people as experts in your research. This thought is not new, and there is a growing body of literature related to participatory autism research (e.g. Fletcher-Watson et al., 2019; Keating, 2021). Although I am a fan of participatory methods, I am not writing this reflection to suggest that all autism researchers need to take this approach. And, I am also not talking about tokenistic engagement in research. I take solace that my autistic graduate students, colleagues, and friends seem to think I am doing okay as a non-autistic autism researcher. However, what I appreciate professionally the most from these relationships is the unique insights, perspectives, and ideas that these friends and colleagues provide. Historically, autism research has privileged academic expertise over lived experience (Griffith et al., 2012; Pellicano et al., 2014). Authentically engaging with autistic people as a non-autistic autism researcher acknowledges their lived experience—a perspective that I do not have, respects requests from the autistic community to be more actively engaged in autism research (Canadian Autism Spectrum Disorders Alliance, 2016; Collis, 2021; Gotham et al., 2015; Nicolaidis et al., 2011), and I have found, makes for better work. Non-autistic autism researchers can and often do wonderful work, but need to learn alongside and from, not for, autistic people.

Maybe my process of grappling with my role as a non-autistic autism researcher is the best thing that could have happened to my research and teaching. It has led me to engage in a process of reflexivity that has changed the way I approach my work and has invigorated a passion to continue on this tough and vulnerable, but highly rewarding, path. I, like many scholars, strive for a world that is truly inclusive of everyone, including embracing the experiences that autistic people bring to the table, which likely offer a different perspective and expertise than me and other non-autistic autism researchers. This type of authentic engagement means having a respected voice at the table. I know that I can bring my research expertise and experience to the table as part of the autism community, while embracing the expertise of the autistic community to truly enact an inclusive space and become the most effective non-autistic autism researcher.
